# Aware but unprepared: the impact of climate change on healthcare workers and service delivery in Africa - a scoping review

**DOI:** 10.3389/fpubh.2025.1693703

**Published:** 2026-01-16

**Authors:** Adelaide Lusambili, Britt Nakstad, Sharon Ochieng, Isioma Igweike, Babatope O. Adebiyi, Sadiq Bhanbhro, Ogechukwu Igweike, Julian Natukunda

**Affiliations:** 1 NextGen for Earth, Nairobi, Kenya; 2 Environmental Health and Governance Centre, School of Business, Africa International University, Nairobi, Kenya; 3 Department of Paediatrics and Adolescent Health, University of Botswana, Gaborone, Botswana; 4 United Lincolnshire Hospitals NHS Trust, Boston, United Kingdom; 5 Section of Rheumatology, Department of Paediatrics, Cumming School of Medicine of Calgary, Calgary, AB, Canada; 6 Centre for Applied Health and Social Care Research, Sheffield Hallam University, Sheffield, United Kingdom; 7 Faculty of Biology, Medicine and Health, School of Medical Science, The University of Manchester, Manchester, United Kingdom; 8 Centre for Tropical Medicine and Global Health, Nuffield Department of Medicine, University of Oxford, Oxford, United Kingdom

**Keywords:** healthcare workers, healthcare professionals, health service delivery, climate change, climate-health impacts, healthcare systems, Africa

## Abstract

**Introduction:**

Climate change is an urgent global crisis, placing a growing strain on health systems and overwhelming healthcare workers’ ability to respond. Africa is especially vulnerable due to its limited resources and infrastructure. Healthcare workers face climate impacts directly, yet their preparedness is poorly understood. This scoping review assessed how climate change affects healthcare workers and service delivery across the continent.

**Methods:**

A scoping review methodology was followed. A systematic literature search was conducted across six electronic databases, including Scopus, CINAHL, Dimensions, PubMed, Web of Science, and Google Scholar. Additional studies were identified via hand searching. Eligible studies included primary research on healthcare professionals’ perceptions, preparedness, and the systemic challenges climate change poses. They were included if published between 2005 and July 2025, conducted in Africa, and written in English. Data were extracted and synthesised to identify common themes and gaps in the current understanding and response to climate-related health issues.

**Results:**

This scoping review synthesised evidence from 18 studies conducted across 17 African countries—including Ghana, Nigeria, South Africa, Namibia, Ethiopia, Kenya, Egypt, Uganda, the Democratic Republic of Congo, Zimbabwe, Sudan, Rwanda, Zambia, Botswana, Malawi, Somalia, and Burkina Faso. The review included 10 quantitative, 5 qualitative, and 3 mixed-methods studies examining healthcare workers’ perceptions, preparedness, and experiences in addressing climate-related health challenges. Results show that healthcare workers in Africa recognise rising climate-related health problems, including vector- and heat-related diseases, respiratory conditions, and malnutrition. However, they feel unprepared to address these challenges, citing insufficient training and inadequate infrastructure. Heavy patient loads contribute to stress and burnout, while gaps in knowledge about causes and adaptation limit prevention. At the facility level, resource shortages and weak climate-health policies further constrain effective and sustainable responses.

**Conclusion:**

Climate change is intensifying healthcare demands, straining limited resources, and burdening health professionals. Targeted policies, resilient infrastructure, effective surveillance systems, and comprehensive training programs are needed to enhance service delivery, reduce strain, and build resilience against climate-related health impacts.

**Systematic review registration:**

https://osf.io/s82uq/.

## Introduction

Climate change is an urgent global crisis, with evidence indicating that Africa will experience more severe impacts than other regions, largely due to its lower adaptive capacity and greater reliance on climate-sensitive sectors such as agriculture and water resources (1). This vulnerability extends to the healthcare sector, where the effects of climate change are already being felt. Research shows that sub-Saharan Africa (SSA) will experience the highest and increasing burden of climate-sensitive diseases, such as malaria, dengue, diarrhoeal diseases, and malnutrition (2, 3). The increased incidence of climate-sensitive diseases adds to the workload of healthcare workers (HCWs), stretching already limited resources and exacerbating burnout (4, 5).

Healthcare facilities struggle to provide care during extreme weather events like flooding, which disrupts health service delivery (6). In other studies, extreme heat has been reported to reduce the efficiency and well-being of HCWs, particularly with little access to cooling facilities, leading to increased stress, absenteeism, and decreased productivity (7, 8). In recent decades, Africa has seen frequent severe weather events, including flooding, rising temperatures, hurricanes, and worsening air pollution. The region’s health systems are already under significant strain, characterised by weak infrastructure, a brain drain of healthcare workers, and the challenge of delivering adequate care to an ageing population and a rapidly growing youth demographic (9). Thus, extreme events linked to climate change disproportionately affect those in rural and underserved areas, including the urban poor and temporary settlements, along with healthcare facilities and workers in these locations, contributing to growing inequalities (10). This strain on health systems from climate change impacts further reduces patient care quality, stretching already limited resources and exacerbating burnout (4, 5, 11, 12).

Primary health care is key to improving national and community responses to climate change and migration (13, 14), and healthcare workers are central to caring for the sick. Therefore, HCWs must be alert and prepared to address these growing challenges and play a key role in strengthening health systems. Research increasingly recommends that healthcare providers engage in discussions on preventing and managing climate-related health impacts (15). Across much of the continent, evidence on how healthcare workers (HCWs) understand and experience climate change remains scarce. Yet we know from other parts of the world that climate extremes—heatwaves, floods, droughts, and storms—can take a heavy toll on those at the frontlines of care. These climate extremes strain HCWs physically and emotionally, and disrupt their ability to provide essential health services when communities need them most. This review aimed to examine healthcare workers’ (HCWs) knowledge and perceptions of climate change (CC), and how CC influences the delivery of health services. It also aimed to assess the preparedness of health systems and the capacity of HCWs to respond effectively to climate-related extremes and associated challenges. Finally, the review sought to identify evidence gaps to guide future research, policy development, and the design of interventions that strengthen climate-resilient health systems and support the wellbeing of HCWs in the African context.

## Methods

### Step 1. Identifying the research question (JBI—PCC)

Given that evidence on this topic remains limited and fragmented, we adopted a scoping review approach to systematically map the available literature, describe the scope and nature of existing research, and identify key gaps to inform future studies and policy responses. The review followed the methodological framework outlined by the Joanna Briggs Institute (JBI) for conducting scoping reviews (16, 17) and adhered to the guidelines of the Preferred Reporting Items for Systematic Reviews and Meta-Analyses extension for Scoping Reviews (PRISMA-ScR) (18).

**Figure 1:**
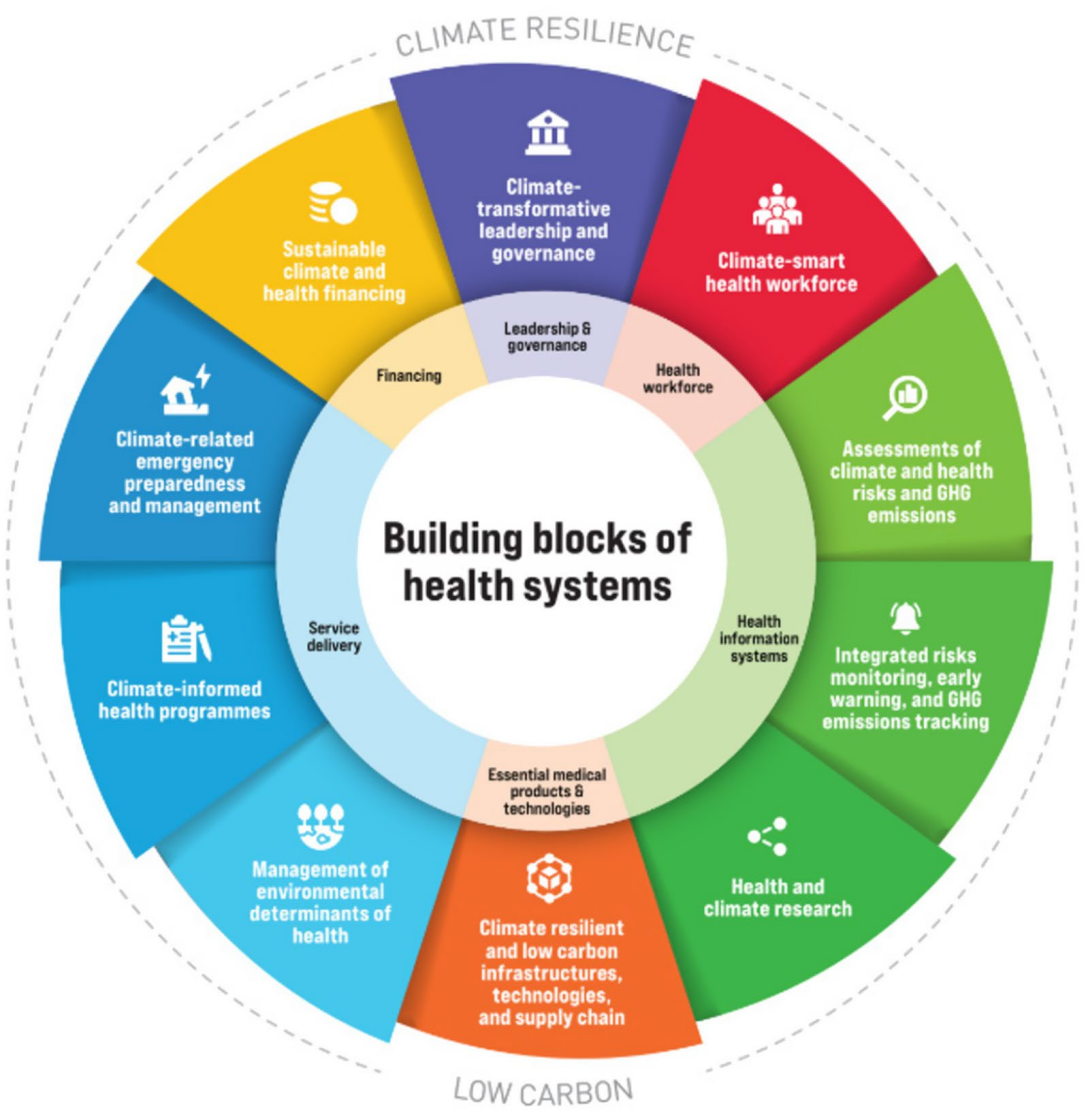
WHO operational framework for climate resilient and low carbon health systems.

The review question was structured using the Population–Concept–Context (PCC) framework, where the population comprised healthcare workers of all cadres in Africa; the concept focused on the impacts of climate change and climate-related hazards such as heatwaves, floods, and storms on health service delivery and healthcare workers’ well-being; and the context encompassed African health systems across all regions. The review was guided by the World Health Organization (WHO) Operational Framework for Building Climate-Resilient and Low-Carbon Health Systems (Figure 1) (19), which informed the interpretation of findings and identification of gaps in preparedness, service delivery, and workforce well-being.

The JBI and PRISMA-ScR methodologies provide a structured approach for mapping evidence on complex and emerging topics, ensuring scientific rigor, transparency, and the identification of research gaps. Recent reviews by Tesfaye (20) and Vandenberg (21) further demonstrate that these frameworks are effective for synthesizing diverse evidence from multiple sources.

### Step 2. Identifying relevant studies

A systematic literature search was conducted across six electronic databases: Scopus, CINAHL, Dimensions, PubMed, Web of Science, and Google Scholar. In addition, a manual search was conducted in the African Journal Online (22) and organisational websites, from February 20th, 2024, to July 31st, 2025.

The search for relevant literature was conducted in collaboration with the librarians from the University of Botswana (23). First, an initial search was conducted in two databases to identify relevant topics and initial keywords, MeSH/index terms. These informed the development of a comprehensive search strategy that included both controlled vocabulary [i.e., Medical Subject Headings (MeSH) and free-text terms related to climate change, HCWs, and health impacts, informed by previously published literature]. Initially, one database was searched to identify relevant topics and MeSH terms, which informed the final search strategy and Table 1. This refined search strategy was uniformly applied across the six databases to extract relevant literature. Additional studies were identified via a hand search using the terms keywords “climate change and healthcare,” “climate change and healthcare workers,” and “impact” or “effects” to identify articles not indexed in the selected databases. The detailed search strategy for all databases is provided in Table 1.

**Table 1:** Summary of search terms (MeSH and free-text keywords).

Database	Controlled vocabulary/subject headings	Free-text keywords and synonyms (by Concept)
PubMed	“Healthcare personnel” [MeSH]; “climate change” [MeSH]; “global warming” [MeSH]; “natural disasters” [MeSH]; “floods” [MeSH]; “cyclonic storms” [MeSH]; “Africa” [MeSH]; “Africa South of the Sahara” [MeSH]	Health workforce: “healthcare providers,” “health workers,” “nurses,” “midwives,” “community health workers,” “doctors,” “pharmacists,” “physiotherapists,” “clinical social workers.” Climate change: “climate changes,” “global warming,” “heatwaves,” “flooding,” “wildfires,” “storms,” “hurricanes.” Geographic: “Africa,” “Sub-Saharan Africa,” “Southern Africa,” and all African country names.
CINAHL	“Healthcare personnel+”; “climate change+”; “global warming+”; “natural disasters+”; “floods+”; “cyclonic storms+”; “Africa+” (CINAHL headings)	Health workforce: “health professionals,” “community health workers,” “nurses,” “midwives.” Climate: “climate change,” “global warming,” “natural disasters,” “heatwaves,” “flooding.” Geographic: “Sub-Saharan Africa,” “Southern Africa,” and country names.
Web of Science	Keyword search using “Topic” field (no standardized thesaurus). Key concepts: health workforce, climate change, Africa.	“Healthcare personnel,” “health workers,” “nurses,” “doctors,” “climate change,” “global warming,” “natural disasters,” “floods,” “hurricanes,” “Africa,” “Sub-Saharan Africa,” and African countries.
Scopus	Title–Abstract–Keyword (TITLE-ABS-KEY) search using three main concepts: health workforce × climate change × Africa.	“Healthcare providers,” “health professionals,” “nurses,” “midwives,” “climate change,” “flooding,” “heatwaves,” “extreme weather,” “Sub-Saharan Africa,” “Southern Africa,” and individual country names.
Google Scholar	Keyword search using thematic clusters (no controlled vocabulary). Concepts: health workforce, climate change, Africa.	“Health workers,” “nurses,” “doctors,” “climate change,” “extreme weather,” “natural disasters,” “Africa,” “Sub-Saharan Africa,” and African countries.

### Step 3. Study selections (exclusion and inclusion criteria)

The review considered only primary research studies (including qualitative, quantitative, mixed methods, and context-specific/occupational studies). We focused on primary research studies to map systematically gathered evidence on the impact of climate change on healthcare workers in Africa. This approach allows us to understand the scope and nature of existing research and pinpoint research gaps. Studies were included if they were published between 1st January 2005 and 31st July 2025, conducted in Africa, and written in English. Although climate change has been recognised as a health concern since the early 1990s (24), its status as a global health priority was reinforced by WHO Resolution WHA61.19 in 2008 (25). Review studies indicate a notable increase in research on climate change and health from 2015 onwards (3, 9), yet evidence from Africa remains limited and unevenly distributed. Selecting 2005 as the start date allows us to capture the emergence of primary research over the past two decades, track trends, and identify gaps, particularly regarding the effects on healthcare professionals.

**Figure 2:**
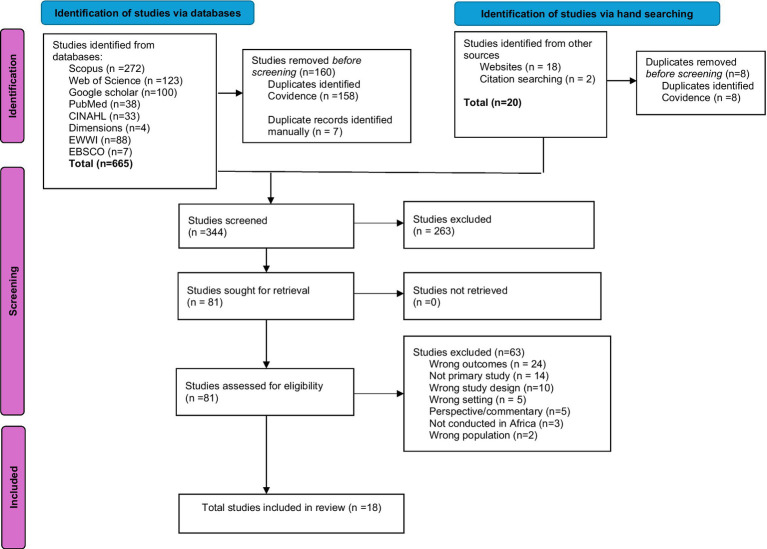
PRISMA flow chart of study selection.

Included in the review were full-text articles with primary research and discussions on the impact of climate change on healthcare workers’ delivery of services and the well-being of both patients and HCWs. Also included were studies focusing on the quality of care, occupational risks or resources, and those narrating experiences (challenges and opportunities) faced by HCWs working in various settings such as African rural and urban areas, humanitarian contexts, cities, and coastal regions. Studies were excluded if they did not focus on HCWs and climate change, were published before 2005, or were not in English. Additionally, grey literature, review papers, book reviews, conference papers, commentaries, perspectives, editorials, and studies conducted in high-income countries were excluded. The review process is summarised in the PRISMA flow chart (Figure 2).

### Selection and data collection process

Identified studies were uploaded into Covidence, a systematic review management system, assessed for duplicates before undergoing title-abstract and full-text screening. Eight reviewers (four early-career and four senior researchers) worked in pairs to co-screen articles according to the eligibility criteria during the title and abstract screening. Early-career researchers were paired with senior researchers throughout all phases of the research process. In the full-text review stage, each article was independently reviewed by two reviewers, who provided reasons for inclusion or exclusion. Reference lists of selected articles were screened to identify additional studies not captured in the databases.

Data extraction was conducted using a pre-designed template, with two reviewers extracting data per article and a third reviewer (senior reviewer) resolving discrepancies to achieve consensus.

### Step 4. Charting the data (data extraction)

Data extraction was conducted using a pre-designed data charting form template (Table 2). The lead reviewer (AL) developed a data extraction/charting form that was reviewed and agreed upon by the team. The tool was pre-tested using three randomly selected papers; the group completed the list collaboratively and amended as necessary. Data were extracted for the following areas: (1) study title, (2) location, (3) study population, (4) method of data collection, (5) sample size and duration, (6) key findings including interventions, and (7) themes summarising the findings. Two reviewers independently extracted data per article, and a third reviewer (senior reviewer) performed a final check to resolve discrepancies to achieve consensus. Each reviewer independently conducted data extraction, with a third reviewer performing a final check to resolve discrepancies. To streamline the charting process, an online Google form was utilised to collect all necessary information.

**Table 2:** Characteristics of the studies included in the scoping review.

		Author(s), Year	Country(s)	Study objective	Participants, sample size	Type of study (methods)	Key findings
1		Opoku et al. (2021) (26)	Ghana, Nigeria, South Africa, Namibia, Ethiopia, Kenya.	Assessing vulnerabilities to climate change, its impact on human health, and preparedness to cope with these health hazards.	Health professionals from government agencies, higher institutions, non-governmental agencies, and research institutions. Sample size: 122	Quantitative, cross-sectional survey and literature review.	Health professionals recognised the increasing impact of climate change on health, particularly in relation to climate-induced diseases, and anticipated that these challenges would continue to escalate. They also reported inadequate preparedness, citing a shortage of adequately trained healthcare professionals, insufficient health delivery systems, and limited resources to effectively address climate-related uncertainties.
2		Manga et al. (2022) (28)	South Africa	Evaluating healthcare professionals' knowledge and perspectives on climate change.	Health care workers (doctors, nurses, medical students, and allied health professionals). Sample size: 234	Quantitative, survey.	The majority of participants (81%) were uncertain whether their healthcare facilities had implemented or planned to implement changes in response to climate change. Over half (79%) believed that their facilities should be more proactive, and 93% anticipated that climate change would adversely affect healthcare workers, their communities, and patients.
3		Mahmoud et al. (2023) (30)	Egypt	Exploring the effect of climate change on health and critical care nurses’ practice.	Nurses working in ICU and emergency units. Sample size: 84	Quantitative, interviews.	Over half of the nurses expressed awareness of, and concern about, the future impacts of climate change on upcoming generations and nursing practice, particularly regarding illnesses linked to poor air quality. Most nurses perceived climate change as harmful to patients, recognising its potential to cause physical, mental, and even violent harm.
4		Scheerens et al. (2021) (27)	Uganda Nigeria, DRC, Uganda, Kenya, Zimbabwe, SA, Sudan, Rwanda, Zambia, Botswana, Malawi, Somalia	Exploring family physicians' perceptions of the interconnections between climate change, migration, and health, with insights into building capacity across sectors to create climate-resilient and migration-inclusive health systems.	Family physicians identified in a conference in Uganda. Sample size: 30	Qualitative, focus group discussions.	Participants identified various categories of interactions within the nexus, including migration-related health impacts. They reported challenges faced by host health facilities in accommodating incoming migrants, citing a lack of integration with refugee health programs. The brain drain of health workers, driven by poor working and living conditions, further weakens workforce capacity and morale, leading to a decline in the effectiveness of health services. Intersectoral capacity-building opportunities include strengthening governance structures and improving training and working conditions for health professionals.
5		Sithole et al. (2021) (41)	Zimbabwe	Understanding how well-equipped health facilities manage and mitigate the climate-related challenges, with specific focus on malnutrition and related health issues.	Healthcare workers. Sample size: 108	Quantitative, multi-cluster assessment tool.	Findings indicated that the health delivery system in Zimbabwe was not adequately prepared to respond to the drought conditions. Most health workers were insufficiently equipped to manage acute malnutrition, nutrition surveillance was weak, and there were notable deficiencies in the stocks of essential nutritional and anthropometric resources.
6		Hussey et al. (2020) (29)	Ghana	Examining the capacities and preparedness of public health professionals to manage climate-related health risks and emergencies.	Public health professionals/health service providers. Sample size: 119 (Survey: 99; Interviews: 20)	Mixed methods, interviews and surveys.	Health professionals were not adequately trained on how climate change impacted health, even though they recognised it as a threat. About 95% recognised climate change as a serious threat that altered disease outbreaks. Despite this awareness, preparedness was lacking. Many professionals incorporated climate health information into their work, yet fewer than 10% were directly involved in researching climate change-related health risks.
7		Nsengiyumva et al. (2020) (31)	Rwanda	Exploring nurses' and midwives' awareness of climate change and perceptions of potential effects on neonatal health in Rwanda.	Nurses and midwives who work in neonatal units. Sample size: 184	Quantitative, cross-sectional survey.	Findings revealed a significant gap in awareness about climate change among the surveyed individuals, with the majority lacking basic knowledge of climate change, its causes, and its impacts on health, particularly for adults and neonates. There was also a notable deficiency in understanding the necessary mitigation and adaptation measures, as well as personal and professional responsibilities related to climate change. Fewer nurses and midwives were aware of established guidelines and tools for integrating climate change strategies into health practices. However, many were unsure how climate change could affect newborn outcomes, such as low birth weight, infections, and respiratory diseases.
8		Kadio et al. (2024) (43)	Burkina Faso	Investigating women's perceptions of heat impacts on their physical and mental health, and their social and economic activities.	Pregnant women, healthcare workers and community members. Sample size: 91	Qualitative, in-depth interviews, key informant interviews and focus group discussions.	Despite exploring mothers’ experiences with heat exposure, it became evident that healthcare services often failed to provide adequate care or information to pregnant women due to a limited understanding of the health implications of heat during pregnancy. This lack of awareness among health workers regarding heat-related issues led to insufficient responses to women’s needs. For instance, healthcare workers were observed misdiagnosing heat-related skin problems as chickenpox. Overall, healthcare staff struggled to provide accurate information and quality care due to gaps in knowledge and awareness.
9		Dos Santos et al. (2019) (36)	South Africa	Addressing and improving urban and community living conditions by identifying shortages in clinical skills and services in specific settlements.	Health care workers. Sample size: 8	Qualitative, key informant interviews.	Findings reveal varied perceptions regarding the impact of climate change on health conditions. Specific heat conditions noted were heat rash, influenza, respiratory and cardiovascular issues, diarrheal and heatstroke. While there is a recognition of certain health issues being exacerbated by climate change, such as respiratory and heat-related illnesses, there is also a variation in the extent to which different health workers perceive these changes as linked to climate effects.
10		Lister et al. (38)	South Africa	Investigating healthcare professionals’ knowledge, attitudes, practices, and barriers to environmental sustainability.	Healthcare professionals. Sample size: 118 (survey: 100; FGDs: 18)	Mixed methods, survey and focus group discussions.	Findings showed that while health professionals were generally aware of environmental sustainability and its importance for human health, they lacked the practical and theoretical knowledge to fully implement practices such as waste reduction and recycling. This lack of knowledge, combined with limited financial resources, time constraints, and inadequate policies and infrastructure, hindered progress. Despite these challenges, most participants believed that environmental sustainability was crucial for the health of both current and future generations. They felt that healthcare professionals should lead in this area, serving as role models for their communities. Education was viewed as a key solution to closing the knowledge gap, alongside addressing financial limitations.
11		Mousa et al. (2024) (32)	Egypt	Assessing nurses’ knowledge and practices regarding climate change in Egypt.	Nursing staff. Sample size: 213	Qualitative, questionnaires and self- assessed checklist.	Findings revealed significant gaps in knowledge and practice that hindered effective healthcare responses to climate change. The majority of nurses demonstrated correct but incomplete knowledge of the negative consequences of climate change (64.8%) and acknowledged its impact on human health (63.4%), plant production (55.9%), and animal production (46%). However, 85.9% of nurses did not perceive a relationship between climate change and the health of individuals in their communities, nor did they associate it with the emergence of new diseases or symptoms. Regarding adaptive practices, 77% reported increasing their fluid intake during periods of high temperature, and 9.9% used cooling devices such as fans or air conditioners for extended periods. A larger proportion (77%) coped with heat by wearing loose cotton clothing. Nonetheless, engagement in broader environmental health practices was limited. Most nurses did not participate in recycling or public awareness initiatives related to climate change, despite reporting appropriate waste disposal and some energy-saving behaviours.
12		Momen et al. (2024) (33)	Egypt	Determining family medicine physicians’ attitudes toward climate change and its impact in a family medicine setting in Egypt.	Family medicine residents and staff physicians. Sample size: 127	Quantitative, descriptive cross-sectional survey.	The study found that although 64% of physicians recognised that climate change was affecting their patients’ health– mainly through mental health issues, allergies, dehydration, and heat stroke–only 17% felt confident discussing it with their patients. Many expected cases of dehydration and heat-related illness to increase over the next 10 to 20 years, while some anticipated a slight decline in mental health impacts. Although most physicians (71%) agreed that climate change was relevant to primary care, only 31% believed it should be openly discussed with patients. Interestingly, only 9.6% felt well-informed about how climate change affected health, even though 48% reported feeling comfortable giving advice. Thishighlighted a clear gap between physicians’ perceived confidence and their actual knowledge.
13		Ashour et al. (2024) (37)	Egypt	Assessing the maternity nurses’ competencies regarding the potential consequences of heat exposure on mothers and newborns in the context of climate change.	Maternity nurses in the obstetrics and gynaecology departments. Sample size: 50	Quantitative, descriptive cross-sectional survey.	The study found that most maternity nurses had a fair understanding of how climate change and heat could affect pregnant women and newborns, with an average knowledge score of 54%. However, 92% were not applying this knowledge in their daily work. Few participated in training, followed hospital guidelines, or took steps to help patients manage heat-related risks. Most nurses obtained their information from social media and television. The study also showed that nurses with greater knowledge were more likely to take action if they received appropriate support. Overall, the study concluded that although maternity nurses possessed a reasonable level of knowledge about the health risks posed by heat exposure amid climate change, their actual practices were unsatisfactory. This disparity between knowledge and implementation highlighted systemic barriers such as lack of training, institutional support, and awareness of actionable practices.
14		Atta et al. (2024) (34)	Egypt	Exploring the relationship between climate anxiety, environmental attitudes, and job engagement among academic nursing staff in Egypt.	Academic nursing staff. Sample size: 359	Quantitative, descriptive, cross-sectional survey.	The findings suggested that climate change was subtly influencing healthcare workers, particularly in terms of how they felt and performed in their roles. Most participants were urban-based women aged 30–40, and around 43% believed their health conditions—mainly cardiovascular, respiratory, or water-borne illnesses—were linked to climate change. Nurses who reported higher levels of climate anxiety also tended to have stronger environmental concerns, yet they felt slightly less motivated and connected to their work. Notably, the different dimensions of job engagement—physical, emotional, and cognitive—were strongly and positively correlated, indicating that these aspects tended to rise or fall together. The study emphasised the need for interventions to address climate anxiety and promote positive environmental attitudes to enhance job engagement and overall well-being among nursing professionals.
15		Abdelaziz et al. (2024) (35)	Egypt	Comparing nurses’ knowledge, skills, and attitudes regarding climate change and its effects on children from hospitals in two provinces in Egypt.	Nursing care providers in paediatric units. Sample size: 336	Quantitative, cross-sectional comparative survey.	The study examined how well paediatric nurses at two hospitals in Egypt understood and responded to climate change and its effects on children’s health. Although most nurses had many years of experience, the majority had not received training on climate change. Nurses at Al Azhar Hospital demonstrated stronger knowledge, better skills, and a more positive attitude than those at Beni-Suef. For instance, more Al Azhar nurses knew how climate change could cause asthma, diarrhoea, and mental stress in children. They were also more likely to take action, such as educating parents or participating in hospital initiatives to address climate-related health risks. The study found that nurses with greater knowledge were better equipped to provide assistance.
16		Lister et al. (2025) (39)	Namibia	Identifying healthcare professionals’ knowledge, attitudes, practices, perceived barriers, and educational needs regarding environmental sustainability in healthcare, in order to inform context-specific interventions.	Healthcare professionals. Sample size: 71	Quantitative, descriptive, cross-sectional survey.	Namibian healthcare professionals demonstrated strong awareness of climate change’s health impacts and a willingness to act, yet few worked in settings with sustainability policies. Common eco-friendly practices existed, but barriers such as limited knowledge, time, resources, and policies hindered progress. Most (96.9%) expressed interest in training, clear guidelines, and collaborative efforts to promote greener healthcare.
17		Siya et al. (2025) (42)	Uganda	Exploring health workers’ perspectives on the intersection of health and climate change, with emphasis on malaria.	Health service providers across 24 health facilities. Sample size: 69	Qualitative, semi-structured interviews.	Healthcare staff across Mount Elgon recognised climate change as a growing challenge to their work, citing unpredictable rains, prolonged droughts, extreme heat, and strong winds. They reported higher incidences of malaria in high-altitude areas, increasing respiratory and gastrointestinal infections, and the emergence of new pests affecting people, crops, and livestock. Floods and impassable roads disrupted care, increased workloads, and strained resources. Many linked these changes to long-term climate shifts, viewing climate variability as part of the broader climate change affecting health and livelihoods.
18		Razak et al. (2025) (40) BN: deleted Abdul as not last name and not according to ref	Ghana	Assessing Ghanaian radiographers’ awareness of sustainability in radiography and strategies for improvement.	Radiographers. Sample size: 172	Quantitative, cross-sectional survey.	Nearly half of the radiographers (47.7%) strongly agreed that climate change was a serious societal concern, indicating clear awareness of the issue. Many suggested ways to support sustainability in their work, including more training (33.7%), collaborating with environmental experts (37.2%), and the development of clear guidelines and policies (29.1%). They also expressed strong interest in implementing sustainable practices. These findings highlighted a clear opportunity to incorporate sustainability-focused education into radiography training and ongoing professional development, helping to translate awareness into practical environmental stewardship.

### Step 5. Data analysis

A content thematic analysis of the charted extracted data was undertaken to understand the impacts of climate change on the health workforce and health service delivery. An iterative, inductive approach was used to identify concepts, emergent themes, and characteristic themes, which were then discussed and refined by the team to determine the key topic areas.

### Step 6. Consultation with stakeholders

The consultation stage, which involves engaging stakeholders to validate and contextualise findings, was not conducted in this review. This is acknowledged as a limitation; however, the findings will be shared with policymakers and public health practitioners through targeted dissemination and presentations after publication to inform future policy and practice.

## Results

### Study selection

Our search yielded 685 records, of which 665 were identified from databases and 20 from other sources (Table 2). After removing 168 duplicates, 517 unique records were screened. Of these, 344 records underwent title and abstract screening, excluding 263 records which did not align with our scope. The remaining 81 records underwent full-text screening for eligibility, resulting in the exclusion of 63 records for reasons such as different outcomes (*n* = 24), not being primary studies (*n* = 14), inappropriate study design (*n* = 10), unsuitable setting (*n* = 5), commentary pieces (*n* = 5), being conducted outside Africa (*n* = 3), or different populations (*n* = 2). Ultimately, 18 studies met the inclusion criteria, forming the evidence base for this review. The study selection process is summarised in the PRISMA flow chart (Figure 2).

### Study characteristics

The review identified 18 studies across Africa, with the largest number conducted in Egypt (*n* = 6), followed by South Africa (*n* = 3) and Ghana (*n* = 2). There were five single-country studies conducted in Uganda, Namibia, Rwanda, Zimbabwe, and Burkina Faso (*n* = 5). Two multi-country studies were identified, being Opoku et al. (26), spanning Ghana, Nigeria, South Africa, Namibia, Ethiopia, and Kenya, and Scheerens et al. (27), which included Uganda among several other countries.

Ten studies focused on healthcare workers’ awareness, knowledge, and preparedness for climate change impacts on health (27–37). Five studies explored nursing practice in specific contexts, including intensive and critical care (30), maternity care (38), paediatrics (34), academic nursing staff (35), and climate anxiety among nursing students (32). Three addressed environmental sustainability in healthcare systems (39–41). Two studies investigated direct climate–health linkages in communities (41, 42) and two examined migration and vulnerable populations (27, 43).

The papers of the 18 included studies used different designs and methodologies; 11 employed quantitative designs, primarily descriptive or cross-sectional surveys. Five studies used qualitative approaches, such as interviews and focus group discussions, while two adopted a mixed-methods design integrating surveys with qualitative components.

The most frequently cited climate hazards included rainfall-related flooding, rising temperatures, drought, and worsening heat impacts. Despite a broad search covering papers published between 1st January 2005 and 31st July 2025, all included papers were recent and published between 2019 and 2025.

The characteristics and emerging themes from the studies included in this scoping review are summarised in Tables 2, 3.

**Table 3:** Summary findings and description of main themes identified, including examples from literature.

Themes identified	Description	Examples from literature
Healthcare professionals recognise the impact of climate change on health (*n* = 13)	Climate change is causing an increase in health issues, including vector- and heat-related diseases, respiratory and cardiovascular problems, and malnutrition. Health professionals are aware of these risks, especially in climate-prone regions.	Opoku et al. (26); Scheerens et al. (27); Manga et al. (28); Mahmoud et al. (30); Hussey et al. (29); Dos Santos et al. (36); Sithole et al. (41); Mousa et al. (32); Momen et al. (33); Ashour et al. (37); Lister et al. (39); Siya et al. 2025 (42); and Abdul Razak et al. (40).
Climate change impact on patients, patient care and service delivery (*n* = 9)	Climate change is posing significant challenges to health service delivery and patient care, with studies highlighting increasing patient numbers that strain health services and reduce the effectiveness and quality of service delivery.	Opoku et al. (26); Manga et al. (28); Mahmoud et al. (30); Scheerens et al. (27); Sithole et al. (41); Hussey et al. (29); Mousa et al. (32); Momen et al. (33); and Siya et al. (42).
Unprepared and unresponsive health systems at facility level (*n* = 10)	The health sector and health facilities’ insufficient preparedness for climate change challenges—including inadequate training, weak delivery and surveillance systems, and limited resources—heavily affect the response to climate impacts on health and healthcare professionals.	Opoku et al. (26); Hussey et al. (29); Mahmoud et al. (30); Scheerens et al. (27); Nsengiyumva et al. (31); Sithole et al. (41); Manga et al. (28); Mousa et al. (32); Ashour et al. (37); and Lister et al. (39).
Mental health impacts of climate change on healthcare workforce (*n* = 5)	Healthcare professionals experience stress and burnout due to increasing patient loads. Most studies ‌report growing mental and emotional strain among healthcare workers, particularly in under-resourced settings.	Opoku et al. (26); Manga et al. (28); Scheerens et al. (27); Hussey et al. (29); and Atta et al. (34).
Inadequate knowledge and information and the unclear role of healthcare workers in addressing climate-health-related impacts (*n* = 15)	Healthcare professionals lack knowledge about the causes and impacts of climate change, leading to misdiagnosis of heat-related conditions and insufficient awareness of mitigation and adaptation measures. As a result, they tend to focus on treating illness rather than recognising how climate change affects community health.	Nsengiyumva et al. (31); Kadio et al. (43); Lister et al. (38); Opoku et al. (26); Manga et al. (28); Dos Santos et al. (36); Scheerens et al. (27); Sithole et al. (41); Hussey et al. (29); Mahmoud et al. (30); Mousa et al. (32); Momen et al. (33); Ashour et al. (37); Abdelaziz et al. (35); and Lister et al. (39).
Inadequate policies and resources in the health sector (*n* = 6)	Studies reveal inadequate health sector policies and resources to address climate change. These gaps hinder environmental sustainability practices such as waste reduction and recycling, highlighting the need for improved policies and stronger resources.	Lister et al. (38); Kadio et al. (43); Sithole et al. (41); Manga et al. (28); Lister et al. (39); and Abdul Razak et al. (40).

**Figure 3:**
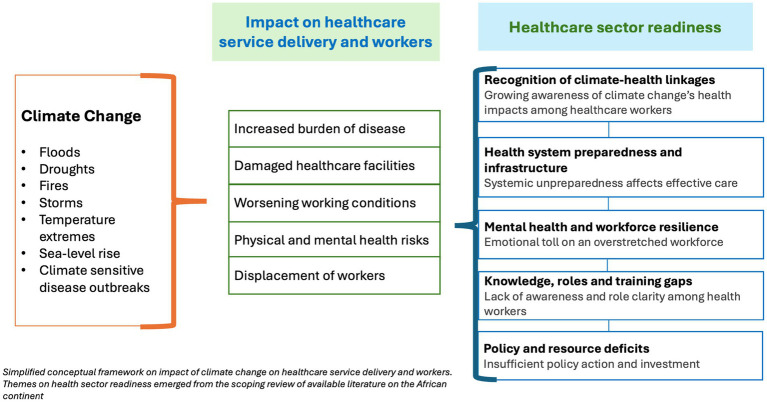
Conceptual framework -study’s findings.

The conceptual framework in Figure 3.

### Healthcare professionals recognise the impact of climate change on health

The reviewed papers consistently found that HCWs recognise the impacts of climate change on health and believe climate change will exacerbate the trend and burden of disease, including vector-, water-, food- and airborne illnesses as well as mental health consequences in the region (27–41). An increase in climate-related health issues, such as respiratory diseases, heat-related illnesses, among others was highlighted (27, 29, 30, 34, 36, 38). Opoku and colleagues found that malaria and other vector-borne diseases were most prevalent in both Ghana and Nigeria (26). In a study from Egypt (35), the majority of nurses did not perceive a connection between climate change and the health of individuals in the local community, nor did they associate climate change with the emergence of new diseases. Further, although physicians recognised that climate change affects their patients’ health, only a few were confident discussing climate change with their patients (33) or using knowledge in their daily practices (32). Most HCWs, although aware of the impact of climate change on health, wished for clear guidelines, more training, and efforts to create a greener healthcare (37, 40). Furthermore, effects were particularly observed in vulnerable communities (41), in critical care nursing practices with regard to patient outcomes (30), and in relation to migration and population displacement (28). In South Africa, nurses recognised that the epidemic of Listeria infectious disease was strongly linked to rising temperatures and water scarcity (28). Additionally, climate change-induced droughts exacerbated malnutrition (41), along with an overall increased burden of diseases (30).

### Unprepared and unresponsive health systems at the facility level

Our review indicates that health systems in SSA countries are largely unprepared and unresponsive to the increasing challenges brought about by climate change. Healthcare workers reported that infrastructure is often insufficient, with a lack of space for isolating patients, no air conditioners, and no or limited sources of running water for infection control and cleanliness; resources which are needed to manage additional strain from climate-related health impacts (27, 29, 30, 33–35, 37, 38). In ten countries, HCWs felt that unpreparedness was related to issues such as insufficient human resources, inadequate healthcare delivery systems to manage climate impacts, and a general lack of priority given to climate-related issues (27, 29–35). Nurses in Egypt could not link climate change to community health or new diseases (35). Further, in Egypt, engagement in broader environmental health practices was limited (35). This was observed even though public awareness initiatives on climate change were in place, and participants reported appropriate waste disposal and some energy-saving behaviours. Adaptive practices were limited, with most relying on simple measures like drinking more water or using fans (35). Manga and colleagues highlighted that HCWs in South Africa expressed the need for healthcare facilities to do more to match the increased demand for services due to climate-induced health issues (28). Similarly, another study found that critical care nurses often reported their facilities as unprepared for the surge in patients with climate-related illnesses, such as heat stroke or respiratory issues (30). These findings were echoed in studies conducted in Zimbabwe and Ghana, where health systems were also found to have insufficient seating space and a shortage of doctors, dieticians, and psychologists to attend to increasing numbers of climate-sensitive patients (29, 41). The lack of preparedness extends to the health system’s ability to meet the needs of refugees or displaced populations and migrants (28).

### Mental health impacts of climate change on the healthcare workforce

Although not extensively discussed, the included studies emphasised the significant adverse impacts of climate change that imposes on the mental health of HCWs. These adverse effects stem largely from their inability to effectively address the climate-related implications for their patients, which often is due to a lack of infrastructure and knowledge. HCWs frequently reported burnout and emotional stress, particularly those facing increased workloads, working in under-resourced settings, or caring for refugees and displaced populations (28, 29). Similarly, a study by Scheerens and colleagues highlighted physicians’ growing anxiety about the mental well-being of both HCWs and patients (27). In a study from Egypt, climate anxiety was prevalent among nurses, which was linked to their feelings and job performance, and nurses highlighted the need for interventions to address climate anxiety and promote positive environmental attitudes for improved well-being (35). In Ghana, when asked about how to address the challenges, HCWs expressed doubts about their ability to handle the mental and emotional demands, particularly those in under-resourced settings (29).

### Climate change impact on patients, patient care and service delivery

Our review highlights the growing impact of climate change on patients and the delivery of patient care, emphasising an urgent concern within the healthcare sector as more patients experience climate-related health issues. An Egyptian study showed that gaps in practice hindered effective healthcare responses to climate change (35). In the Momen et al. study, most HCWs recognised climate change as relevant to primary care, yet only a third believed it should be discussed with patients (33). In a study conducted in Ghana, nearly half of the radiographers viewed climate change as a serious concern and called for more training, collaboration with experts, and clear sustainability guidelines (40).

Findings highlighted that the unprepared health facilities greatly impede HCWs’ ability to handle the increasing demands on patient care, resulting in inadequate care (27, 29, 30). For example, they explained that lack of preparedness, such as outdated surveillance systems and insufficient resources, exacerbated the effects of vector-borne diseases and heat-related illnesses. This unpreparedness limits the healthcare system’s ability to predict and plan a correct diagnosis and treat these conditions. Unpreparedness may lead to misdiagnoses, delayed treatments, longer wait times, and an overall reduction in the quality of care (29–31). These challenges were more pronounced in areas affected by drought, where malnutrition was more prevalent, especially among children (41). Furthermore, the strain on health services with increasing patient numbers, coupled with limited resources, was associated with the loss of HCWs through migration and brain drain, further reducing the capacity for effective healthcare delivery (28).

### Inadequate knowledge and information, and the unclear role of healthcare workers in addressing climate-health-related impacts

The literature highlights a notable deficiency in awareness and knowledge among HCWs regarding the specific health impacts and the current and future associations of climate change and health, along with inadequate training on climate-related health risks (31, 33–36, 40). For example, in Rwanda, findings showed low perceptions of the association between climate change and neonatal health risks (31). Other studies consistently identified significant gaps in understanding and preparedness among HCWs, particularly concerning the broader health implications of climate change and the needs of displaced populations (33–36, 40). Furthermore, HCWs reported insufficient awareness and preparedness about the nutritional impacts of climate change. In Burkina Faso, HCWs were reported to misdiagnose heat-related illnesses (43).

According to Kadio et al. (43), healthcare services in Burkina Faso failed to provide adequate care or climate information and showed limited understanding of the health implications of heat during pregnancy. Mousa et al. (32) reported significant gaps in knowledge and practice among nurses that hindered effective healthcare responses to climate change in daily work. Similarly, in Momen et al. (34), physicians agreed that climate change is relevant to primary care, but it was not often discussed with patients. However, experienced nurses in Egyptian hospitals were better equipped to respond and more likely to educate parents or participate in hospital efforts to address climate-related health risks (35). In Namibia, healthcare professionals recognised the health impacts of climate change and were willing to act, but few worked in settings with sustainability policies (39).

Mahmoud and colleagues found that over half of the nurses believed they had no personal role and responsibility in addressing climate change, instead viewing it as the duty of health organisations and national leaders (30). This is attributed to HCWs being preoccupied with routine and often urgent healthcare provision, rarely giving them opportunities for training on health impacts on climate change (33). Moreover, Hussey and colleagues pointed out the underutilisation of available resources, with few facilities that had access to environmental health practitioners, as these visited once every four months (29). Overall, studies reported that HCWs focused on treating illnesses instead of recognising and offering targeted interventions to support adaptation to how climate change affects community health. Also, most HCWs were unclear about their role in addressing climate change, its effects on health and patient care, and their involvement in supporting adaptation efforts (30, 33–36, 40).

### Inadequate policies and resources in the health sector

Studies emphasised the lack of comprehensive and actionable climate-health policies that address the health impacts of climate change, including policy gaps that hinder resource coordination and the delivery of quality health services, affecting health outcomes (27, 29, 30, 37, 40). Dos Santos and colleagues emphasised the need for policymakers to adopt statutory, technological, educational, and behavioural approaches, such as telemedicine and enhanced training for health professionals, noting that telemedicine could mitigate disruptions in healthcare delivery during extreme weather events (37). Other studies pointed to the necessity of policies that foster knowledge sharing, capacity building, and adaptation strategies, including managing heat risks by critical improvements related to HCWs’ resilience and closing gaps in service delivery (27, 29, 30, 40). Additionally, some studies identified weak surveillance and early warning systems as contributing to poor preparedness for climate-sensitive diseases, leaving health systems reactive rather than proactive (27, 29, 30). To address this, they advocated for policy frameworks aimed at strengthening disease surveillance through partnerships with various stakeholders and training HCWs at all levels of governance.

## Discussion

Our findings stress the vulnerability of healthcare systems and HCWs in SSA, as well as their limited capacity to adapt to the effects of climate change. Included studies show that HCWs are aware and recognise the rising impacts of climate change on human health, exacerbating the disease burden in the SSA region. In addition, healthcare roles and responsibilities are becoming more demanding, yet health systems remain largely unprepared, resulting in inadequate patient care and increased stress and burnout among workers. The review identified gaps in the understanding of links between climate change and specific disease conditions, limited knowledge about relevant policies, inadequacies in existing policies, and uncertainty among HCWs regarding their role in supporting adaptation efforts.

HCWs recognise that climate change is occurring and impacting the lives of those they care for, anticipating that it will intensify the burden of climate-sensitive diseases such as vector-borne, water-borne, food, heat, and air-borne illnesses, if no action is taken to mitigate its effects. Conditions such as heat stress, heatstroke, malnutrition, and respiratory illnesses were frequently mentioned alongside malaria and diarrhoea, indicating a growing awareness of heat-related and air-borne diseases in the region. Further, health impacts from extreme weather events, such as heavy rainfall and rising temperatures, significantly increase patient loads at health facilities, placing strain on resources and service delivery. Similar findings were reported from reviews and studies among HCWs worldwide, showing higher awareness of heat-, vector-, and airborne diseases (44–47). Although there is increasing acknowledgement of the climate-related impacts, HCWs continue to express uncertainty about their role in responding to these impacts or how to support health adaptation in their work (46, 47).

All included studies identified significant gaps in HCWs’ knowledge of the health impacts of climate change, with variations across specialities. A key issue was the lack of adequate training on climate-related health risks, which hindered their ability to diagnose and manage issues like heat risks in pregnant women and neonates, as seen in Burkina Faso and Rwanda (31, 43). There was also limited awareness in addressing malnutrition and linking rising malaria cases and flooding to broader environmental changes. Few HCWs were familiar with guidelines that integrate climate change into health practice or with necessary adaptation measures. Several studies reported similar findings, noting that while HCWs are motivated to address climate change, they are not actively involved in initiatives to combat it. The majority emphasise the need to bridge the knowledge-action gap through more training (48), continuous medical education (49), interdisciplinary collaboration, and the integration of climate change into healthcare curricula (50–53).

Our review found that healthcare systems were unprepared and unresponsive to climate change challenges, with insufficient infrastructure, a lack of space for isolating patients, inadequate air conditioning, and unreliable water sources for infection control. These deficiencies strain healthcare delivery and negatively impact HCWs, especially in regions with displaced populations, where most facilities lack planned emergency resources. Evidence also suggests that some facilities had access to environmental health practitioners but never utilised their services, despite their potential to enhance HCWs’ skills or provide additional support (30). Our findings align with previous studies about the vulnerability of healthcare systems to climate impacts (53, 54).

Healthcare workers reported increased vulnerability to mental and emotional stress, particularly in under-resourced settings. Physicians expressed concerns about the mental health of both patients and HCWs, as they face increased stress and burnout from managing rising patient loads, which negatively impacts the quality of care. These challenges are largely attributed to their inability to effectively address climate-related health issues due to poor infrastructure and insufficient knowledge. The findings align with growing concerns and reports linking climate change and mental health risks, and these are not different for HCWs globally (55, 56). However, there is limited research on interventions aimed at reducing the impact of climate change on HCWs and health systems, especially in Africa, and actionable mental health interventions are yet to be incorporated in health policies in the region. To develop suitable interventions and support strategies, evidence-based protocols and further research are needed to explore the health challenges HCWs face because of climate change. This underscores the urgent need to integrate mental health support into welfare programs.

Lastly, the review revealed a significant gap in comprehensive and actionable climate-health policies, which obstructs resource coordination and the delivery of quality health services. Weak surveillance and early warning systems were identified as key barriers to preparedness for climate-sensitive diseases, leading to reactive health systems. Additionally, many HCWs reported having little to no knowledge of climate-health policies at the facility level. Findings agree with current literature, which suggests that only 21 countries in Africa have developed health national adaptation plans (HNAPs), and seven of these have HNAPs that are less than five years old (57). Countries supported by international agencies are developing climate-change adaptation plans to guide future interventions (58–60). Further, findings of a 2023 study on national disease surveillance systems in 14 African states indicated that while there are relatively functional surveillance systems for malaria, other diseases are lagging, and available surveillance systems do not integrate environmental data (61). Other studies emphasise the need for improved and integrated surveillance systems (58, 60, 61). Strengthening disease surveillance and training HCWs across all governance levels is crucial for enhancing preparedness and response. Additionally, policies should focus on promoting knowledge sharing, capacity building, and adaptation strategies to manage heat risks and build resilience effectively.

Given that healthcare workers across Africa recognise the rising health threats from climate change but remain uncertain about how to respond, there is an urgent need to embed climate–health training within health education and integrate adaptation strategies into national health planning. Ministries of Health, universities, and partners should strengthen health system resilience and establish clear policies that empower healthcare workers to lead climate preparedness efforts.

Most studies recommend increased government investment in health infrastructure, disaster response funding (53), and strategies like big data and telemedicine (36, 54, 62). Research into structural working and living conditions in rural and remote settings to boost resiliency and improve benefits for HCWs to fill vacant healthcare positions could improve rural health service delivery (49). The health sector must prioritise strengthening infrastructure and resources to better prepare for climate-related challenges. Key areas include improving facilities for increased patient loads, ensuring reliable water and energy supplies, boosting human resources, and enhancing infection control and emergency preparedness.

Some studies have explored ways climate change topics could be introduced into existing programs (51), including framing a climate change perspective in problem-based learning cases (standardised reporting/sharing of experiences and initiatives), discussing climate issues on clinical rotations, and research (50). Also, WHO has provided guidance, including a communication toolkit, to help HCWs effectively convey the health risks of climate change (52). More effective learning approaches are needed that move beyond the traditional top-down “lecture and learn” model. Health systems should integrate early warning alerts for climate-sensitive diseases and extreme weather into routine care. Real-time information will help healthcare workers prepare and act quickly to protect patients. At the same time, behavioural adaptation training—through regular drills, guidance, and climate–health education—should empower staff to respond effectively and build a proactive culture of climate resilience.

### Limitations

There is a limited amount of primary data regarding the effects of climate change on healthcare professionals within the African context. Despite conducting a thorough search across various databases, only 18 publications from 17 countries were included, which may restrict the applicability of the conclusions drawn to the heterogeneous regions of Africa. Furthermore, the inclusion of studies with small sample sizes may lower the statistical power for quantitative studies and limit causal attributions. The exclusion of grey literature, commentaries, and non-English sources may have further restricted the breadth of the data, overlooking valuable insights from areas where climate change impacts may be underreported. Gaps in data, particularly regarding the mental health impacts on HCWs, further limit the scope of this review, highlighting the need for more focused research in this area. This review adhered to the methodological framework outlined by the Joanna Briggs Institute (JBI) for scoping reviews. However, Stage 6, consultation with stakeholders, was not undertaken, primarily due to time and logistical constraints. The absence of this phase limits the integration of experiential and policy perspectives that could have enriched the contextual interpretation of the findings. This review protocol will be registered retrospectively.

## Conclusion

This scoping review highlights the significant challenges that climate change presents to healthcare systems and HCWs in Africa. It identifies critical gaps in awareness and preparedness among HCWs, exacerbated by inadequate training, unclear roles, and the lack of comprehensive climate-health policies. Recommendations emphasise the need for targeted policy development to enhance the resilience of healthcare systems. Key steps include strengthening infrastructure, improving surveillance systems, and providing comprehensive training for HCWs. Additionally, fostering a sense of responsibility among HCWs is vital for proactive engagement in climate-related health issues. Addressing these challenges will not only enhance patient care but also reduce the mental and emotional strain on HCWs, ultimately improving overall healthcare delivery.

## Data Availability

The datasets presented in this study can be found in online repositories. The names of the repository/repositories and accession number(s) can be found in the article/Supplementary material.

## References

[1] Niang I Ruppel OC Abdrabo MA Essel A Lennard C Padgham J . Africa In: Barros VR Field CB Dokken DH Mastrandrea MD Mach KJ Bilir TE , editors. Climate change 2014: impacts, adaptation, and vulnerability. Part B: regional aspects. Cambridge: Cambridge University Press (2014). 1199–265.

[2] World Health Organization. Health and climate change: country profile 2015: South Africa. Geneva: WHO; 2015.

[3] Romanello M McGushin A Di Napoli C Drummond P Hughes N Jamart L . The 2021 report of the lancet countdown on health and climate change. Lancet. (2021) 398:1619–62. doi: 10.1016/S0140-6736(21)01787-6, 34687662 PMC7616807

[4] Thomson MC Mason S Platzer B Mihretie A Omumbo J Mantilla G . Climate and health in Africa. Earth Perspect. (2014) 1:17. doi: 10.1186/2194-6434-1-17

[5] Mohtady Ali H Ranse J Roiko A Desha C. Healthcare workers’ resilience toolkit for disaster management and climate change adaptation. Int J Environ Res Public Health. (2022) 19:12440. doi: 10.3390/ijerph191912440, 36231739 PMC9564616

[6] Salam A Wireko AA Jiffry R Ng JC Patel H Zahid MJ . Impact of natural disasters on healthcare and surgical services in LMICs. Ann Med Surg Lond. (2023) 85:3774–7. doi: 10.1097/MS9.0000000000003774 37554857 PMC10406090

[7] Xiang J Bi P Pisaniello D Hansen A. Health impacts of workplace heat exposure: an epidemiological review. Ind Health. (2014) 52:91–101. doi: 10.2486/indhealth.2012-0145, 24366537 PMC4202759

[8] Boonstra WJ Hanh TTH. Adaptation to climate change as social-ecological trap: tam Giang lagoon, Vietnam. Environ Dev Sustain. (2015) 17:1527–44. doi: 10.1007/s10668-014-9603-6

[9] Watts N Amann M Arnell N Ayeb-Karlsson S Belesova K Boykoff M . The 2019 lancet countdown report. Lancet. (2019) 394:1836–78. doi: 10.1016/S0140-6736(19)32596-6 31733928 PMC7616843

[10] Kjellstrom T Briggs D Freyberg C Lemke B Otto M Hyatt O. Heat, human performance, and occupational health. Annu Rev Public Health. (2016) 37:97–112. doi: 10.1146/annurev-publhealth-032315-021740, 26989826

[11] Lugten E Hariharan N. Strengthening health systems for climate adaptation and health security. Health Secur. (2022) 20:435–9. doi: 10.1089/hs.2021.0171, 35904944 PMC9595646

[12] Chang A Gundling K. Innovating patient care in the era of climate change. J Climate Change Health. (2023) 13:100250. doi: 10.1016/j.joclim.2023.100250

[13] Rasanathan K Evans TG. Primary health care, the declaration of Astana and COVID-19. Bull World Health Organ. (2020) 98:801–8. doi: 10.2471/BLT.20.272252, 33177777 PMC7607474

[14] Lokotola CL Mash R Naidoo K Mubangizi V Mofolo N Schwerdtle PN. Climate change and primary health care in Africa: a scoping review. J Climate Change Health. (2023) 11:100229. doi: 10.1016/j.joclim.2023.100229

[15] Chersich MF Wright CY. Climate change adaptation in South Africa: the role of the health sector. Glob Health. (2019) 15:22. doi: 10.1186/s12992-019-0479-8 PMC6423888 30890178

[16] Peters MDJ Marnie C Tricco AC Pollock D Munn Z Alexander L . Updated methodological guidance for scoping reviews. JBI Evid Synth. (2020) 18:2119–26. doi: 10.11124/JBISRIR-D-19-00360 33038124

[17] Peters MDJ Godfrey C McInerney P Khalil H Larsen P Marnie C . Best practice guidance for scoping review protocols. JBI Evid Synth. (2022) 20:953–68. doi: 10.11124/JBIES-21-00192 35102103

[18] Tricco AC Lillie E Zarin W O’Brien KK Colquhoun H Levac D . PRISMA-ScR: checklist and explanation. Ann Intern Med. (2018) 169:467–73. doi: 10.7326/M18-0850 30178033

[19] World Health Organization. Operational framework for building climate-resilient and low-carbon health systems. Geneva: WHO (2023).

[20] Tesfaye AH Prior J McIntyre E. Impact of climate change on health workers: a scoping review. J Public Health. (2025) 33:1–12. doi: 10.1007/s10389-025-02418-z, 41395571

[21] Vandenberg SY Chircop A Sedgwick M Scott D. Nurses’ perceptions of climate-sensitive vector-borne diseases: a scoping review. Public Health Nurs. (2023) 40:468–84. doi: 10.1111/phn.13163 36760037

[22] African Journals Online (AJOL). Available online at: https://www.ajol.info/index.php/ajol (Accessed 10 October 2025).

[23] Hadie SNH. Abc of a scoping review: a simplified JBI scoping review guideline. Educ Med J. (2024) 16:185–97. doi: 10.21315/eimj2024.16.2.7

[24] World Health Organization. Climate change and human health: Risk and responses. Geneva: WHO (2003).

[25] World Health Organization. Resolution WHA61.19: Global strategy on health, environment and climate change. Geneva: WHO (2008).

[26] Opoku SK Leal Filho W Hubert F Adejumo O. Climate change and health preparedness in Africa: six-country analysis. Int J Environ Res Public Health. (2021) 18:4672. doi: 10.3390/ijerph18094672, 33925753 PMC8124714

[27] Scheerens C Bekaert E Ray S Essuman A Mash B Decat P . Family physician perceptions of climate change, migration, health and healthcare in SSA. Int J Environ Res Public Health. (2021) 18:6323. doi: 10.3390/ijerph18126323 34207979 PMC8296126

[28] Manga A Dartchiev D Variava E. Healthcare and climate change: a south African health professionals’ perspective. Wits J Clin Med. (2022) 4:173–80. doi: 10.18772/26180197.2022.v4n3a8

[29] Hussey LK Arku G. Health systems preparedness for climate risks: Ghana. Clim Dev. (2020) 12:170–82. doi: 10.1080/17565529.2019.1605284

[30] Mahmoud FH Mahmoud BH. Effect of climate change on health and critical care nurses’ practice. Egypt J Hosp Med. (2023) 90:1149–55. doi: 10.21608/ejhm.2023.312546

[31] Nsengiyumva R Mukarubayiza MR Murekatete C Meharry P. Climate change associated with neonatal health risks: Rwandan nurses/midwives’ perceptions. Rwanda J Med Health Sci. (2020) 3:261–72.

[32] Mousa NM Elshair EH Elsawy MM. Climate anxiety and nurses’ practice toward climate change (Bahariya oasis, Egypt). Egypt Nurs J. (2024) 21:111–20.

[33] Momen M Allam I Basyoni NI. Family medicine physician attitudes towards climate change and health in Egypt. Egypt J Community Med. (2024) 42:84–9.

[34] Atta MHR Zoromba MA El-Gazar HE Loutfy A Elsheikh MA El-Ayari OS . Academic nursing staff: environmental attitude and job engagement. BMC Nurs. (2024) 23:133. doi: 10.1186/s12912-024-01612-5 38378543 PMC10880327

[35] Abdelaziz SF Elzeiny A Fouda NM Shahin MAH Alabed HH Loutfy A. Nurses’ knowledge, skills and attitudes on climate change impacts on children’s health in Egyptian hospitals. Salud Cienc Tecnol. (2024) 4:e4567. doi: 10.56294/sct2024e4567

[36] Dos Santos M Howard D Kruger P Banos A Kornik S. Climate change and healthcare sustainability in the Agincourt sub-district, South Africa. Sustainability. (2019) 11:496. doi: 10.3390/su11020496

[37] Ashour ES El-Razek AA Abd El-Salam AA. Maternity nurses’ competences regarding heat exposure in climate change. Egypt J Health Care. (2024) 15:1570–83.

[38] Lister HE Mostert K Botha T van der Linde S van Wyk E Rocher SA . South African healthcare professionals’ knowledge, attitudes and practices on environmental sustainability. Int J Environ Res Public Health. (2022) 19:10121. doi: 10.3390/ijerph191610121 36011760 PMC9408692

[39] Lister HE Mostert K Ramkilawon G Oelschig C Ntiyane O Richardt E . Namibian healthcare professionals’ knowledge, attitudes, and practices on sustainability among Namibian healthcare professionals. Int J Environ Res Public Health. (2025) 22:751. doi: 10.3390/ijerph22050751 40427866 PMC12111493

[40] Abdul Razak W Takyi C Ofori-Manteaw BB. Exploring sustainability in radiography: Ghanaian radiographers. Radiography. (2025) 31:102952. doi: 10.1016/j.radi.2025.102952, 40233647

[41] Sithole Z Nyadzayo T Kanyowa T Mathieu J Kambarami T Nemaramba M . Rapid nutritional assessment in Zimbabwe’s drought. Pan Afr Med J. (2021) 40:113. doi: 10.11604/pamj.2021.40.113.27186 34887987 PMC8627146

[42] Siya A Lukwa AT Chemutai F Mutai N Chiwire P. Health workers’ perspectives on climate change and health in Kween District, Uganda. J Clim Change Health. (2025) 23:100463. doi: 10.1016/j.joclim.2025.100463 PMC12851208 41647369

[43] Kadio K Filippi V Congo M Scorgie F Roos N Lusambili A . Extreme heat, pregnancy and women’s well-being in Burkina Faso: an ethnography. BMJ Glob Health. (2024) 8:e014230. doi: 10.1136/bmjgh-2023-014230 PMC10897842 38382997

[44] Kotcher JE Maibach E Miller J Campbell E Alqodmani L Maiero M . Views of health professionals on climate change and health: a multinational survey. Lancet Planet Health. (2021) 5:e316–23. doi: 10.1016/S2542-5196(21)00053-X 33838130 PMC8099728

[45] Sanderson R Galway LP. Perceptions of climate change and climate action among climate-engaged health professionals in northern Ontario. J Clim Change Health. (2021) 3:100025. doi: 10.1016/j.joclim.2021.100025

[46] Salas RN Malina D Solomon CG. Prioritizing health in a changing climate. N Engl J Med. (2019) 381:773–4. doi: 10.1056/NEJMp1906035, 31433926

[47] Quitmann C Sauerborn R Danquah I Herrmann A. Climate change mitigation is a hot topic, but not when it comes to hospitals. J Med Ethics. (2023) 49:204–10. doi: 10.1136/jme-2022-108524 35459742 PMC9985738

[48] Sorensen CJ Fried LP. Defining roles and responsibilities of the health workforce to respond to the climate crisis. JAMA Netw Open. (2024) 7:e241435. doi: 10.1001/jamanetworkopen.2024.1435, 38517435

[49] Lemery J Balbus J Sorensen C Rublee C Dresser C Balsari S . Training clinical and public health leaders in climate and health. Health Aff. (2020) 39:2189–96. doi: 10.1377/hlthaff.2020.01011 33284695

[50] Quinn Griffin MT Alfes CM Chavez F Ea EE Lynn KA Rafferty MA . Incorporating climate change into doctor of nursing practice curricula. J Prof Nurs. (2022) 42:156–61. doi: 10.1016/j.profnurs.2022.07.005 36150855

[51] Brennan ME Madden DL. Including climate change and environmental sustainability in health professional education: a scoping review. J Clim Change Health. (2023) 9:100200. doi: 10.1016/j.joclim.2023.100200

[52] World Health Organization. Communicating on climate change and health: Toolkit for health professionals. Geneva: WHO (2024).

[53] Naser K Haq Z Naughton BD. Impact of climate change on health services in LMICs: a systematised review and thematic analysis. Int J Environ Res Public Health. (2024) 21:434. doi: 10.3390/ijerph21040434, 38673345 PMC11050668

[54] Tsakonas K Badyal S Takaro T Buse C. Rapid review of the impacts of climate change on the health system workforce and implications for action. J Climate Change Health. (2024) 19:100337. doi: 10.1016/j.joclim.2024.100337 PMC12851351 41647318

[55] Lawrance EL Thompson R Newberry Le Vay J Page L Jennings N. Impact of climate change on mental health and emotional wellbeing: current evidence and implications for policy and practice. Int J Environ Res Public Health. (2022) 19:7974. doi: 10.3390/ijerph19137974, 36165756

[56] Clayton S Karazsia BT. Development and validation of a measure of climate change anxiety. J Environ Psychol. (2020) 69:101434. doi: 10.1016/j.jenvp.2020.101434

[57] World Health Organization. Health National Adaptation Process (HNAP) progress report. Geneva: WHO (2023).

[58] United Nations Environment Programme. Adaptation gap report 2023. Nairobi: UNEP (2023).

[59] African Union Commission. Africa climate change and resilient development strategy and action plan 2022–2032. Addis Ababa: AUC (2022).

[60] World Health Organization Regional Office for Africa. Climate change and health in the African region: Review of evidence and opportunities for action. Brazzaville: WHO AFRO (2023).

[61] Musa M Omoleke SA Shuaib F Okeke I Bolarinwa O . Strengthening disease surveillance and response systems in Africa: a review of gaps and opportunities. BMJ Glob Health. (2024) 9:e013745. doi: 10.1136/bmjgh-2023-013745

[62] Katapally TR Bhawra J. Inverting social innovation to transform health system responses to climate change in the global south. Front Public Health. (2024) 12:1333163. doi: 10.3389/fpubh.2024.1333163, 38803808 PMC11128584

