# Supplementary material for: Aware but unprepared: the impact of climate change on healthcare workers and service delivery in Africa - a scoping review
Source: Front Public Health. 2026 Jan 16;13:1693703. doi: 10.3389/fpubh.2025.1693703 (PMC12855563; doi:10.3389/fpubh.2025.1693703)
Supplement: Supplementary file 2 [file Data_Sheet_2.docx]

**Search strategy**

**Impact of climate change on healthcare workers' delivery of health services in Africa: A scoping review**

**PubMed**

"Healthcare personnel"[Mesh] OR "healthcare providers”[tiab] OR “healthcare professionals"[tiab] OR "health workers"[tiab] OR doctors[tiab] OR physicians[tiab] OR "medical specialists"[tiab] OR nurses[tiab] OR midwives[tiab] OR "community health workers"[tiab] OR "allied health personnel"[tiab] OR dentists[tiab] OR "dental auxiliaries"[tiab] OR paramedics[tiab] OR radiographers[tiab] OR pharmacists[tiab] OR physiotherapists[tiab] OR "physical therapists"[tiab] OR optometrists[tiab] OR "clinical social worker"[tiab] AND "Climate change"[Mesh] OR "climate changes"[tiab] OR "global warming"[Mesh] OR "sea level rise" OR "natural disasters"[Mesh] OR heatwaves[tiab] OR floods[Mesh] OR flooding[tiab] OR "wild fires"[tiab] OR "cyclonic storms"[Mesh] OR "tropical storms"[tiab] OR storms[tiab] OR cyclon*[tiab] OR hurricanes[tiab] AND Africa[Mesh] OR African[tiab] OR Africans[tiab] OR "Africa South of the Sahara"[Mesh] OR "Sub-Saharan Africa"[tiab] OR "Sub-Sahara Africa"[tiab] OR "Sub Saharan Africa" OR "Southern Africa" OR SADC[tiab] OR "Africa, Central"[Mesh] OR "Central Africa"[tiab] OR "Africa, Eastern"[Mesh] OR "East Africa"[tiab] OR "Eastern Africa"[tiab] OR "Africa, Northern"[Mesh] OR "North Africa"[tiab] OR "Northern Africa"[tiab] OR "Africa, Western"[Mesh] OR "West Africa"[tiab] OR "Western Africa"[tiab] OR Algeria[tiab] OR Angola[tiab] OR Benin[tiab] OR Botswana[tiab] OR "Burkina Faso"[tiab] OR Burundi[tiab] OR "Cape Verde"[tiab] OR Cameroon[tiab] OR "Central African Republic"[tiab] OR Chad[tiab] OR Comoros[tiab] OR Congo[tiab] OR "Democratic Republic of the Congo"[tiab] OR "Democratic Republic Congo"[tiab] OR DRC[tiab] OR Congo[tiab] OR "Cote d'Ivoire"[tiab] OR Djibouti[tiab] OR Egypt[tiab] OR Equatorial[tiab] OR Guinea[tiab] OR Eritrea[tiab] OR Swaziland[tiab] OR Eswatini[tiab] OR Ethiopia[tiab] OR Gabon[tiab] OR Gambia[tiab] OR Ghana[tiab] OR Guinea[tiab] OR Guinea-Bissau[tiab] OR Kenya[tiab] OR Lesotho[tiab] OR Liberia[tiab] OR Libya[tiab] OR Madagascar[tiab] OR Malawi[tiab] OR Mali[tiab] OR Mauritania[tiab] OR Mauritius[tiab] OR Morocco[tiab] OR Mozambique[tiab] OR Namibia[tiab] OR Niger[tiab] OR Nigeria[tiab] OR Rwanda[tiab] OR "Sao Tome and Principe"[tiab] OR Senegal[tiab] OR Seychelles[tiab] OR "Sierra Leone"[tiab] OR Somalia[tiab] OR "South Africa"[tiab] OR "South Sudan"[tiab] OR Sudan[tiab] OR Tanzania[tiab] OR Togo[tiab] OR Tunisia[tiab] OR Uganda[tiab] OR Zambia[tiab] OR Zimbabwe[tiab]

**CINAHL**

(MH "Healthcare personnel+") OR (TI "healthcare providers" OR AB "healthcare providers") OR (TI "healthcare professionals" OR AB "healthcare professionals") OR (TI "health workers" OR AB "health workers") OR (TI doctors OR AB doctors) OR (TI physicians OR AB physicians) OR (TI "medical specialists" OR AB "medical specialists") OR (TI nurses OR AB nurses) OR (TI midwives OR AB midwives) OR (TI "community health workers" OR AB "community health workers") OR (TI "allied health personnel" OR AB "allied health personnel") OR (TI dentists OR AB dentists) OR (TI "dental auxiliaries" OR AB "dental auxiliaries") OR (TI paramedics OR AB paramedics) OR (TI radiographers OR AB radiographers) OR (TI pharmacists OR AB pharmacists) OR (TI physiotherapists OR AB physiotherapists) OR (TI "physical therapists" OR AB "physical therapists") OR (TI optometrists OR AB optometrists) OR (TI "clinical social worker" OR AB "clinical social worker") AND (MH "Climate change+") OR (TI "climate changes" OR AB "climate changes") OR (MH "global warming+") OR "sea level rise" OR (MH "natural disasters+") OR (TI heatwaves OR AB heatwaves) OR (MH floods+) OR (TI flooding OR AB flooding) OR (TI "wild fires" OR AB "wild fires") OR (MH "cyclonic storms+") OR (TI "tropical storms" OR AB "tropical storms") OR (TI storms OR AB storms) OR (TI cyclon* OR AB cyclon*) OR (TI hurricanes OR AB hurricanes) AND (MH Africa+) OR (TI African OR AB African) OR (TI Africans OR AB Africans) OR (MH "Africa South of the Sahara+") OR (TI "Sub-Saharan Africa" OR AB "Sub-Saharan Africa") OR (TI "Sub-Sahara Africa" OR AB "Sub-Sahara Africa") OR "Sub Saharan Africa" OR "Southern Africa" OR (TI SADC OR AB SADC) OR (MH "Africa, Central+") OR (TI "Central Africa" OR AB "Central Africa") OR (MH "Africa, Eastern+") OR (TI "East Africa" OR AB "East Africa") OR (TI "Eastern Africa" OR AB "Eastern Africa") OR (MH "Africa, Northern+") OR (TI "North Africa" OR AB "North Africa") OR (TI "Northern Africa" OR AB "Northern Africa") OR (MH "Africa, Western+") OR (TI "West Africa" OR AB "West Africa") OR (TI "Western Africa" OR AB "Western Africa") OR (TI Algeria OR AB Algeria) OR (TI Angola OR AB Angola) OR (TI Benin OR AB Benin) OR (TI Botswana OR AB Botswana) OR (TI "Burkina Faso" OR AB "Burkina Faso") OR (TI Burundi OR AB Burundi) OR (TI "Cape Verde" OR AB "Cape Verde") OR (TI Cameroon OR AB Cameroon) OR (TI "Central African Republic" OR AB "Central African Republic") OR (TI Chad OR AB Chad) OR (TI Comoros OR AB Comoros) OR (TI Congo OR AB Congo) OR (TI "Democratic Republic of the Congo" OR AB "Democratic Republic of the Congo") OR (TI "Democratic Republic Congo" OR AB "Democratic Republic Congo") OR (TI DRC OR AB DRC) OR (TI Congo OR AB Congo) OR (TI "Cote d'Ivoire" OR AB "Cote d'Ivoire") OR (TI Djibouti OR AB Djibouti) OR (TI Egypt OR AB Egypt) OR (TI Equatorial OR AB Equatorial) OR (TI Guinea OR AB Guinea) OR (TI Eritrea OR AB Eritrea) OR (TI Swaziland OR AB Swaziland) OR (TI Eswatini OR AB Eswatini) OR (TI Ethiopia OR AB Ethiopia) OR (TI Gabon OR AB Gabon) OR (TI Gambia OR AB Gambia) OR (TI Ghana OR AB Ghana) OR (TI Guinea OR AB Guinea) OR (TI Guinea-Bissau OR AB Guinea-Bissau) OR (TI Kenya OR AB Kenya) OR (TI Lesotho OR AB Lesotho) OR (TI Liberia OR AB Liberia) OR (TI Libya OR AB Libya) OR (TI Madagascar OR AB Madagascar) OR (TI Malawi OR AB Malawi) OR (TI Mali OR AB Mali) OR (TI Mauritania OR AB Mauritania) OR (TI Mauritius OR AB Mauritius) OR (TI Morocco OR AB Morocco) OR (TI Mozambique OR AB Mozambique) OR (TI Namibia OR AB Namibia) OR (TI Niger OR AB Niger) OR (TI Nigeria OR AB Nigeria) OR (TI Rwanda OR AB Rwanda) OR (TI "Sao Tome and Principe" OR AB "Sao Tome and Principe") OR (TI Senegal OR AB Senegal) OR (TI Seychelles OR AB Seychelles) OR (TI "Sierra Leone" OR AB "Sierra Leone") OR (TI Somalia OR AB Somalia) OR (TI "South Africa" OR AB "South Africa") OR (TI "South Sudan" OR AB "South Sudan") OR (TI Sudan OR AB Sudan) OR (TI Tanzania OR AB Tanzania) OR (TI Togo OR AB Togo) OR (TI Tunisia OR AB Tunisia) OR (TI Uganda OR AB Uganda) OR (TI Zambia OR AB Zambia) OR (TI Zimbabwe OR AB Zimbabwe)

**Web of Science**

"Healthcare personnel" OR "healthcare providers" OR "healthcare professionals" OR "health workers" OR doctors OR physicians OR "medical specialists" OR nurses OR midwives OR "community health workers" OR "allied health personnel" OR dentists OR "dental auxiliaries" OR paramedics OR radiographers OR pharmacists OR physiotherapists OR "physical therapists" OR optometrists OR "clinical social worker" AND "Climate change" OR "climate changes" OR "global warming" OR "sea level rise" OR "natural disasters" OR heatwaves OR floods OR flooding OR "wild fires" OR "cyclonic storms" OR "tropical storms" OR storms OR cyclon* OR hurricanes AND Africa OR African OR Africans OR "Africa South of the Sahara" OR "Sub-Saharan Africa" OR "Sub-Sahara Africa" OR "Sub Saharan Africa" OR "Southern Africa" OR SADC OR "Africa, Central" OR "Central Africa" OR "Africa, Eastern" OR "East Africa" OR "Eastern Africa" OR "Africa, Northern" OR "North Africa" OR "Northern Africa" OR "Africa, Western" OR "West Africa" OR "Western Africa" OR Algeria OR Angola OR Benin OR Botswana OR "Burkina Faso" OR Burundi OR "Cape Verde" OR Cameroon OR "Central African Republic" OR Chad OR Comoros OR Congo OR "Democratic Republic of the Congo" OR "Democratic Republic Congo" OR DRC OR Congo OR "Cote d'Ivoire" OR Djibouti OR Egypt OR Equatorial OR Guinea OR Eritrea OR Swaziland OR Eswatini OR Ethiopia OR Gabon OR Gambia OR Ghana OR Guinea OR Guinea-Bissau OR Kenya OR Lesotho OR Liberia OR Libya OR Madagascar OR Malawi OR Mali OR Mauritania OR Mauritius OR Morocco OR Mozambique OR Namibia OR Niger OR Nigeria OR Rwanda OR "Sao Tome and Principe" OR Senegal OR Seychelles OR "Sierra Leone" OR Somalia OR "South Africa" OR "South Sudan" OR Sudan OR Tanzania OR Togo OR Tunisia OR Uganda OR Zambia OR Zimbabwe

**Scopus**

"Healthcare personnel" OR "healthcare providers" OR "healthcare professionals" OR "health workers" OR doctors OR physicians OR "medical specialists" OR nurses OR midwives OR "community health workers" OR "allied health personnel" OR dentists OR "dental auxiliaries" OR paramedics OR radiographers OR pharmacists OR physiotherapists OR "physical therapists" OR optometrists OR "clinical social worker" AND "Climate change" OR "climate changes" OR "global warming" OR "sea level rise" OR "natural disasters" OR heatwaves OR floods OR flooding OR "wild fires" OR "cyclonic storms" OR "tropical storms" OR storms OR cyclon* OR hurricanes AND Africa OR African OR Africans OR "Africa South of the Sahara" OR "Sub-Saharan Africa" OR "Sub-Sahara Africa" OR "Sub Saharan Africa" OR "Southern Africa" OR SADC OR "Africa, Central" OR "Central Africa" OR "Africa, Eastern" OR "East Africa" OR "Eastern Africa" OR "Africa, Northern" OR "North Africa" OR "Northern Africa" OR "Africa, Western" OR "West Africa" OR "Western Africa" OR Algeria OR Angola OR Benin OR Botswana OR "Burkina Faso" OR Burundi OR "Cape Verde" OR Cameroon OR "Central African Republic" OR Chad OR Comoros OR Congo OR "Democratic Republic of the Congo" OR "Democratic Republic Congo" OR DRC OR Congo OR "Cote d'Ivoire" OR Djibouti OR Egypt OR Equatorial OR Guinea OR Eritrea OR Swaziland OR Eswatini OR Ethiopia OR Gabon OR Gambia OR Ghana OR Guinea OR Guinea-Bissau OR Kenya OR Lesotho OR Liberia OR Libya OR Madagascar OR Malawi OR Mali OR Mauritania OR Mauritius OR Morocco OR Mozambique OR Namibia OR Niger OR Nigeria OR Rwanda OR "Sao Tome and Principe" OR Senegal OR Seychelles OR "Sierra Leone" OR Somalia OR "South Africa" OR "South Sudan" OR Sudan OR Tanzania OR Togo OR Tunisia OR Uganda OR Zambia OR Zimbabwe

**Google Scholar**

"Healthcare personnel" OR "healthcare providers" OR "healthcare professionals" OR "health workers" OR doctors OR physicians OR "medical specialists" OR nurses OR midwives OR "community health workers" OR "allied health personnel" OR dentists OR "dental auxiliaries" OR paramedics OR radiographers OR pharmacists OR physiotherapists OR "physical therapists" OR optometrists OR "clinical social worker" AND "Climate change" OR "climate changes" OR "global warming" OR "sea level rise" OR "natural disasters" OR heatwaves OR floods OR flooding OR "wild fires" OR "cyclonic storms" OR "tropical storms" OR storms OR cyclon* OR hurricanes AND Africa OR African OR Africans OR "Africa South of the Sahara" OR "Sub-Saharan Africa" OR "Sub-Sahara Africa" OR "Sub Saharan Africa" OR "Southern Africa" OR SADC OR "Africa, Central" OR "Central Africa" OR "Africa, Eastern" OR "East Africa" OR "Eastern Africa" OR "Africa, Northern" OR "North Africa" OR "Northern Africa" OR "Africa, Western" OR "West Africa" OR "Western Africa" OR Algeria OR Angola OR Benin OR Botswana OR "Burkina Faso" OR Burundi OR "Cape Verde" OR Cameroon OR "Central African Republic" OR Chad OR Comoros OR Congo OR "Democratic Republic of the Congo" OR "Democratic Republic Congo" OR DRC OR Congo OR "Cote d'Ivoire" OR Djibouti OR Egypt OR Equatorial OR Guinea OR Eritrea OR Swaziland OR Eswatini OR Ethiopia OR Gabon OR Gambia OR Ghana OR Guinea OR Guinea-Bissau OR Kenya OR Lesotho OR Liberia OR Libya OR Madagascar OR Malawi OR Mali OR Mauritania OR Mauritius OR Morocco OR Mozambique OR Namibia OR Niger OR Nigeria OR Rwanda OR "Sao Tome and Principe" OR Senegal OR Seychelles OR "Sierra Leone" OR Somalia OR "South Africa" OR "South Sudan" OR Sudan OR Tanzania OR Togo OR Tunisia OR Uganda OR Zambia OR Zimbabwe
